# SARS-CoV-2-Neutralizing Antibodies

**DOI:** 10.3390/vaccines12111256

**Published:** 2024-11-05

**Authors:** Yawen Liu, Jianhui Nie

**Affiliations:** Division of HIV/AIDS and Sex-Transmitted Virus Vaccines, National Institutes for Food and Drug Control (NIFDC), State Key Laboratory of Drug Regulatory Science, NHC Key Laboratory of Research on Quality and Standardization of Biotech Products, NMPA Key Laboratory for Quality Research and Evaluation of Biological Products, Beijing 102629, China; yawenliu13@163.com

The COVID-19 pandemic, triggered by the SARS-CoV-2 virus, has profoundly and permanently affected many aspects of the world. The COVID-19 vaccination is the most critical strategy to induce a protective immune response and may be the only way to prevent the spread of infection and progression to severe disease and death. The types of COVID-19 vaccines mainly include inactivated vaccines, mRNA vaccines, adenovirus vector vaccines, and protein subunit vaccines [1]. These vaccines are highly effective at preventing severe illness, hospitalization, and death. Neutralizing antibody levels are important indicators of vaccine effectiveness [2]. This Special Issue features the latest scientific and technological advancements in the field of SARS-CoV-2 neutralization assay and vaccine development. It outlines new techniques for neutralization assays and highlights the critical importance of neutralizing antibody evaluation for vaccine and antiviral development.

Vaccine-induced protection depends on a broad humoral immune response and a strong cellular immune response. These responses work together to effectively prevent infection caused by pathogens. The COVID-19 vaccines, which encode the spike (S) protein of SARS-CoV-2, can broadly induce anti-spike antibodies and neutralizing antibodies (nAbs) [3,4]. The humoral immune response plays a key role in antiviral defense. Specifically, the production of specific antibodies in response to SARS-CoV-2 antigens controls viral replication through neutralization [5].

NAbs play a vital role in the prevention and control of COVID-19 infection, providing immune protection to individuals by blocking the virus from entering host cells, reducing viral transmission, enhancing immune clearance, preventing reinfection, and influencing the course of the disease. The following are some common methods for detecting neutralizing antibodies against COVID-19: (1) The first method is virus neutralization tests (VNT), including the classical virus neutralization test (cVNT), and plaque reduction neutralization test (PRNT) [6,7,8]. (2) The second is the surrogate virus neutralization test (sVNT), a protein-based binding assay for detecting neutralizing antibodies (nAbs), offering a simpler and faster alternative. The PremaLabs Diagnostics COVID-19 rapid nAbs test enables large-scale testing. The evaluation of this test kit is based on the use of the international standard for anti-SARS-CoV-2 immunoglobulin (NIBSC 20/136) [9]. In addition, the commercial sVNT microarray quantifies the distinct characteristics of variant-specific neutralizing antibodies for different SARS-CoV-2 variants and Omicron subvariants [10]. The main target antigen of inactivated and recombinant COVID-19 vaccines is the spike (S) protein, which is typically quantified using methods such as ELISA [11,12]. The ELISA method based on Isotope Dilution Tandem Mass Spectrometry (IDMS) can accurately quantify the S protein content of the prototype, Delta, and Omicron strains, enabling quality control for variant-based vaccines and multivalent vaccines against variants. (3) The third method is the Pseudovirus Neutralization Test (pVNT), (4) the fourth is Lateral Flow Assays (LFA), and (5) the fifth is Chemiluminescent Immunoassays (CLIA). The pseudovirus-based neutralization assay quantifies the neutralizing activity of human serum through some reporter genes. This method can also be used to develop antiviral drug molecules and to assess vaccine efficacy [13].

The ongoing evolution and mutation of SARS-CoV-2 underscore the necessity for more potent vaccines or novel COVID-19 vaccination strategies.

Identifying the precise binding epitope of potent neutralizing antibodies is crucial for rational vaccine design and the development of antibody-based therapies. By comparing the differences in sera neutralizability among individuals with acquired immunity, infection-induced humoral immunity appears to establish a more mature and finely selected antibody repertoire. The application of peptide microarray technology to analyze B cell epitopes may facilitate the development and implementation of peptide-based vaccines [14,15,16].

It was proven that heterologous vaccination induces more effective immunogenicity and a longer-lasting antibody response [17]. Based on clinical research results, compared to the homologous vaccination regimen, a booster dose of the mRNA vaccine BNT162b2 after receiving the viral vector vaccine ChAdOx1 induces higher levels of binding and neutralizing antibodies.

Neutralizing antibody testing plays a critical role in research on drug treatments for COVID-19, and is of significant importance in drug development, efficacy evaluation, and monitoring immune responses. In order to effectively respond to the ongoing mutations of the SARS-CoV-2 virus, some novel molecules (molnupiravir, baricitinib, sotrovimab, novel Mabs, etc.) [18] and repurposed drugs (dexamethasone, naproxen, remdesivir, hydroxychloroquine, etc.) have been approved for use [19]. Due to the lack of sufficient clinical data, the efficacy and safety of these novel molecules and repurposed drugs have not been determined.

Adjuvants are defined as various components that enhance the immunogenicity of vaccines when administered in conjunction with vaccine antigens. They include synthetic small-molecule compounds, complex natural extracts, and particulate materials. Clinical trials have shown that they help enhance the intensity, breadth, and durability of the immune response [20,21]. Xu and colleagues have compared the immune effects of inactivated vaccines with different adjuvants in mice. They found that the vaccine group with adjuvants induced higher levels of neutralizing antibodies compared to the group without adjuvants. Furthermore, the levels of neutralizing antibodies and binding antibodies in the adjuvant group showed significant differences, suggesting that adjuvants may also have different mechanisms of action and immunological characteristics [22].

The relationship between vaccine evaluation methods and vaccine development is dynamic and reciprocal, with the processes including preclinical evaluation, clinical trials, accelerated evaluation methods, post-marketing surveillance, immunological evaluation, variant surveillance, and adaptation. Evaluation methods provide critical data that inform and shape the development process, ensuring that vaccines are safe, effective, and able to meet public health needs.
